# Molecular mechanism of anticancer effect of heat shock protein 90 inhibitor BIIB021 in human bladder cancer cell line

**DOI:** 10.1007/s00210-024-02950-x

**Published:** 2024-01-19

**Authors:** Aydemir Asdemir, Aykut Özgür

**Affiliations:** 1 Faculty of Medicine, Department of Urology, Sivas Cumhuriyet University, Sivas, Turkey; 2 Artova Vocational School, Department of Veterinary Medicine, Laboratory and Veterinary Health Program, Tokat Gaziosmanpasa University, Tokat, Turkey

**Keywords:** Bladder cancer, Chemotherapy, HSP90, Chaperone, T24, BIIB021

## Abstract

Bladder cancer is a type of urologic malignancy that exhibits significant morbidity, mortality, and treatment costs. Inhibition of heat shock protein 90 (HSP90) activity has been a promising pharmacological strategy for blocking of bladder cancer pathogenesis. BIIB021 is a next-generation HSP90 inhibitor which interrupts ATP hydrolysis process of HSP90 and inhibits the stabilization and correct folding of client proteins. In current study, we aimed to investigate the molecular mechanism of the anticancer activity of BIIB021 in human bladder cancer T24 cells. Our results revealed that nanomolar concentration of BIIB021 decreased viability of T24 cell. BIIB021 downregulated HSP90 expression in T24 cells and inhibited the refolding activity of luciferase in the presence of T24 cell lysate. PCR array data indicated a significant alteration in transcript levels of cancer-related genes involved in metastases, apoptotic cell death, cell cycle, cellular senescence, DNA damage and repair mechanisms, epithelial-to-mesenchymal transition, hypoxia, telomeres and telomerase, and cancer metabolism pathways in T24 cells. All findings hypothesize that BIIB021 could exhibit as effective HSP90 inhibitor in the future for treatment of bladder cancer patients.

## Introduction

Bladder cancer is an important factor in cancer deaths worldwide and its prevalence is increasing every year, especially in industrialized countries. The risk of bladder cancer is three times higher in men compared to women in the world (Mori et al. 2020; Burger et al. 2013). In recent years, targeted drugs have increased survival rates in bladder cancer patients, but more effective target-specific chemotherapeutics need to be designed in treatment of bladder cancer (Tran et al. 2021; Lenis et al. 2020).

HSP90 is an important molecular chaperone that plays vital roles in the correct folding and stability of various oncogenic client proteins within cells. HSP90 is aberrantly expressed in cancer cells and its interaction between oncogenic client proteins is essential biological process in all phases of tumorigenesis. HSP90 has a conserved N-terminal domain (NTD) that is responsible for the hydrolysis of ATP. The hydrolysis energy of ATP provides conformational change of HSP90, termed the “V-shaped open conformation.” Oncogenic client proteins bind to hydrophobic residues of HSP90 middle domain (MD) with high affinities in the open conformation. Therefore, the prevention of ATPase activity has been an important strategy for cancer drug discovery. To date, the inhibition efficiency of numerous compounds on HSP90 ATPase activity has been investigated in a wide variety of cancer type. There is still no HSP90 inhibitor approved by the FDA as a result of clinical phase studies and therefore, more preclinical data on HSP90 inhibitors are needed in many different cancer types (Özgür and Tutar 2016; Ren et al. 2022; Li et al. 2020; Neckers and Workman 2012; Costa et al. 2020). HSP90 has been considered as a promising biological target in the treatment of bladder cancer. Therefore, the anticancer potentials of HSP90 inhibitors have been extensively investigated on bladder cancer models, but more comprehensive molecular studies are needed (Yoshida et al. 2011; Karkoulis et al. 2010; Chehab et al. 2015).

BIIB021 (also known as CNF2024) (6-chloro-9-((4-methoxy-3,5-dimethylpyridin-2-yl)methyl)-9H-purin-2-amine) is an orally available and fully synthetic inhibitor of HSP90 that selectively interacts with ATP-binding pocket of NTD. A nanomolar concentration of BIIB021 inhibits ATPase activity of HSP90, leading to the degradation and destabilization of oncogenic client proteins and disruption of cancer-related signaling pathways (Lundgren et al. 2009; He and Hu 2018; He et al. 2016). Pre-clinical studies reported that BIIB021 stimulates apoptotic signaling pathways and blocks metastasis and drug resistance mechanisms in a wide variety of cancer types including breast, cervix, leukemia, head and neck, thyroid, lymphoma, and ovarian (Güven and Özgür 2023; Wang et al. 2014a, b; Gopalakrishnan et al. 2013). In clinical studies, anticancer activities of BIIB021 have been evaluated in patients with advanced solid and gastrointestinal stromal tumors (Dickson et al. 2013; Saif et al. 2014).

The current study aims to investigate the molecular mechanisms of anticancer efficiency of BIIB021 on human bladder cancer cell line, namely T24. Our findings indicated that BIIB021 has significant potential for treatment of bladder cancer as a target specific drug.

## Materials and methods

### Materials

BIIB021 was purchased from Adooq BioScience (Irvine, USA). T-24 cell line was obtained from American Type Culture Collection (Manassas, United States). McCoy’s 5a culture medium, trypsin–EDTA, fetal bovine serum (FBS), phosphate buffer saline (PBS), L-glutamine solution, and penicillin-streptomycin solution were supplied from Serana Europe (Pessin, Germany). Colorimetric XTT cell viability kit was from Biological Industries (Kibbutz Beit-Haemek, Israel). Dimethyl sulfoxide (DMSO) and BCA protein assay kit were from SERVA Electrophoresis GmbH (Heidelberg, Germany). RNA isolation and cDNA synthesis kits were from Thermo Scientific (Massachusetts, USA). Human Cancer Pathway Finder™ PCR array (PAHS-033Z) and SYBR green master mix (330500) were from Qiagen (Hilden, Germany). HSP90 ELISA kit was form YL Biont (Shanghai, China). Luciferase was supplied from Sigma Aldrich.

### Cell culture

The cytotoxic activity of BIIB021 was examined against T24 cell line (human urothelial bladder cancer cell line). T24 cells were obtained from a transitional cell carcinoma of the bladder in a female patient. In preclinical research, T24 cell line is extensively used as a grade III human urinary bladder carcinoma model. The cells were grown in L-glutamine containing McCoy’s 5a medium enriched with 1% penicillin–streptomycin and 10% FBS. Cells were cultivated at 37 ºC, 95% humidity, and 5% CO_2_ condition. In exponential stage of growth, the cells were passaged using trypsin–EDTA solution every two to three days.

### Cytotoxicity assay

XTT cell viability kit was applied to detect the cytotoxic effect of BIIB021 in T24 cells. Firstly, 5 × 10^3^ T24 cells were seeded in 96-well plates in 100 μL of McCoy’s 5a medium and incubated for overnight. BIIB021 was supplied in powder form and dissolved in DMSO at a stock concentration of 10 mM. Then, the cells were incubated with BIIB021 for 48 h at concentrations ranging from 100 to 1.562 nM with twofold dilution. After overnight incubation, the XTT kit was applied according to the manufacturer’s instructions. The IC_50_ values of the BIIB021 were calculated with GraphPad Prism 9.0 software.

### Measurement of HSP90 protein expression level

The protein expression level of HSP90 was determined in BIIB021-treated T24 cells comparing with untreated control cells using ELISA test. The cells incubated with IC_50_ value of BIIB021 (16.65 nM) for 48 h. After incubation, BIIB021-treated T24 cell lysate and untreated T24 cell lysate were prepared with RIPA lysis buffer and the human HSP90 ELISA kit was employed according to the manufacturer’s instructions. The alteration of protein expression level of HSP90 was determined in BIIB021 treated T24 cells compared to the untreated cells.

### Luciferase aggregation assay

Luciferase aggregation assay was performed to determine inhibitory effect of BIIB021 on protein folding activity of HSP90 (Karademir and Özgür 2023). In the first step, HSP90 was recombinantly expressed using *E. coli* BL21(DE3) competent cells transformed with the pET-22b(+) cloning vector. The culture was propagated in 1 L selective LB medium supplemented with ampicillin at 37 °C and protein expression was induced with 0.05 mM isopropyl-β-D-thiogalactoside (IPTG) according to the expression-trial results. After induction process for 4 h, the cells were lysed using ultrasonicator instrument and HSP90 was purified by affinity chromatography using Ni–NTA resin. In the second step, luciferase was denatured with urea and diluted in buffer solution (pH:7.4, 50 mM KCl, 5 mM MgCl_2_, 2 mM ATP, 25 mM HEPES, and 5 mM dithiothreitol). BIIB021 (16.65 nM) and T24 cell lysate were added to the mixture and the refolding ratio of denatured luciferase in effect of BIIB021 was calculated spectrometrically at 320 nm.

### PCR array experiment

To identify genes important in the anticancer activity of BIIB021, a gene expression profiling analysis in T24 cells was carried out using the Human Cancer Pathway Finder™ PCR array. Cancer-related and housekeeping genes are listed in Table 1. In this assay, the cells (1 × 10^6^) were seeded into a 25-cm^2^ cell culture flask and the cells were incubated with BIIB021 (16.65 nM) for 48 h. After incubation, total RNA was isolated using a commercial total RNA isolation kit following the manufacturer’s instructions. The amount and purity of isolated RNA was measured by nanodrop spectrophotometer. Then, cDNA was synthesized using a commercial first-strand cDNA synthesis kit, according to the manufacturer’s instructions (1 μg of total RNA was used for cDNA synthesis). PCR array experiment was performed in BioRad CFX96™ (California, United States) instrument using SYBR green master mix with following cycling condition: 1cycle: 95 °C for 10 min, 40 cycles: 95 °C for 15 s and 60 °C for 1 min. The relative expression level of genes was determined with the 2^−ΔΔCt^ method using the cycle threshold (C_t_) values for each gene.

**Table 1:** The list of genes in human Cancer Pathway Finder™ PCR array kit (genes related to cancer)

	1	2	3	4	5	6	7	8	9	10	11	12
A	*ACLY*	*ACSL4*	*ADM*	*ANGPT1*	*ANGPT2*	*APAF1*	*ARNT*	*ATP5A1*	*AURKA*	*BCL2L11*	*BIRC3*	*BMI1*
B	*CA9*	*CASP2*	*CASP7*	*CASP9*	*CCL2*	*CCND2*	*CCND3*	*CDC20*	*CDH2*	*CFLAR*	*COX5A*	*CPT2*
C	*DDB2*	*DDIT3*	*DKC1*	*DSP*	*E2F4*	*EPO*	*ERCC3*	*ERCC5*	*ETS2*	*FASLG*	*FGF2*	*FLT1*
D	*FOXC2*	*G6PD*	*GADD45G*	*GPD2*	*GSC*	*HMOX1*	*IGFBP3*	*IGFBP5*	*IGFBP7*	*KDR*	*KRT14*	*LDHA*
E	*LIG4*	*LPL*	*MAP2K1*	*MAP2K3*	*MAPK14*	*MCM2*	*MKI67*	*NOL3*	*OCLN*	*PFKL*	*PGF*	*PINX1*
F	*POLB*	*PPP1R15A*	*SERPINB2*	*SERPINF1*	*SKP2*	*SLC2A1*	*SNAI1*	*SNAI2*	*SNAI3*	*SOD1*	*SOX10*	*STMN1*
G	*TBX2*	*TEK*	*TEP1*	*TERF1*	*TERF2IP*	*TINF2*	*TNKS*	*TNKS2*	*UQCRFS1*	*VEGFC*	*WEE1*	*XIAP*
H	*ACTB*	*B2M*	*GAPDH*	*HPRT1*	*RPLP0*	*HGDC*	*RTC*	*RTC*	*RTC*	*PPC*	*PPC*	*PPC*

### Pathway analysis

Following PCR array experiment, the correlation between the expression levels of significant 31 genes in T24 cells treated with BIIB021 and cancer signaling pathways was investigated using Enrichr web tool (http://amp.pharm.mssm.edu/Enrichr/) with KEGG (Kyoto Encyclopedia of Genes and Genomes) human gene set library. The performance of the analysis was evaluated by *p*-value and *q*-value. In this analysis, top ten significant pathways and their correlations with up or downregulated genes in BIIB021 treated T24 cells were determined.

### Statistical analysis

This study aims to reveal the anticancer properties of the BIIB21 inhibitor on bladder cancer cells, and this study is not hypothesis-based. Therefore, the determined *p* values were excluded. In pathway analysis with Enrichr web-tool, the *q*-value is an adjusted *p*-value calculated using the Benjamini-Hochberg method for correction of analysis.

## Results

### Cytotoxicity assay

**Fig. 1:**
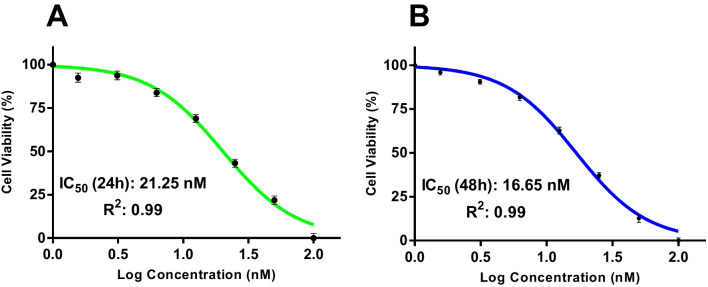
IC_50_ graphs of T24 cells treated with BIIB021 at 24 h (**A**) and 48 h (**B**) (*n* = 3)

To determine BIIB021’s impact on the viability of bladder cancer cells, XTT cell viability assay was carried out to measure the cell inhibition rate of T24 cells treated with varying concentrations of BIIB021 for 48 h. As shown in Fig. 1, BIIB021 decreased the viability of bladder cancer cells in a dose-dependent manner and the IC_50_ values of BIIB021 in T24 cells were calculated as 21.25 nM and 16.65 nM for 24 h and 48 h, respectively.

### HSP90 protein expression level

**Fig. 2:**
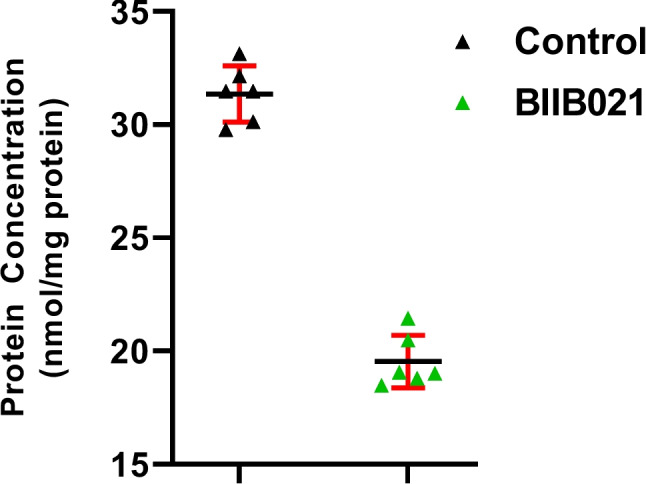
Scatter plots of protein expression level of HSP90 in BIIB021 treated T24 human bladder cancer cells compared with untreated cells (*n* = 6)

The protein level of HSP90 was determined in BIIB021-treated T24 cells by ELISA assay. The results demonstrated that the treatment of T24 cells with IC_50_ value of BIIB21 (16.65 nM) was decreased HSP90 protein level compared with untreated cells (Fig. 2).

### Luciferase aggregation assay

**Fig. 3:**
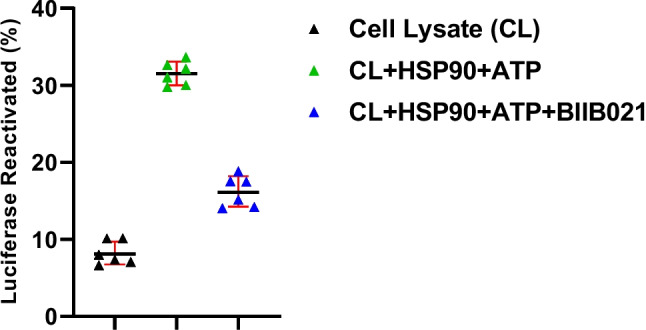
Scatter plots of the refolding activity of denatured luciferase with HSP90 chaperone activity after 48 h in BIIB021 treated T24 human bladder cancer cells (*n* = 6)

The inhibitory effect of BIIB021 on the proper folding and stabilization of proteins by HSP90 chaperone activity was investigated by luciferase aggregation assay. In this assay, the addition of recombinant HSP90 and ATP to the cell lysate increased the refolding rate of denatured luciferase. However, BIIB021 efficiently decreased luciferase refolding level by about 0.6-fold in the presence of recombinant HSP90 and ATP (Fig. 3).

### PCR array experiment

**Fig. 4:**
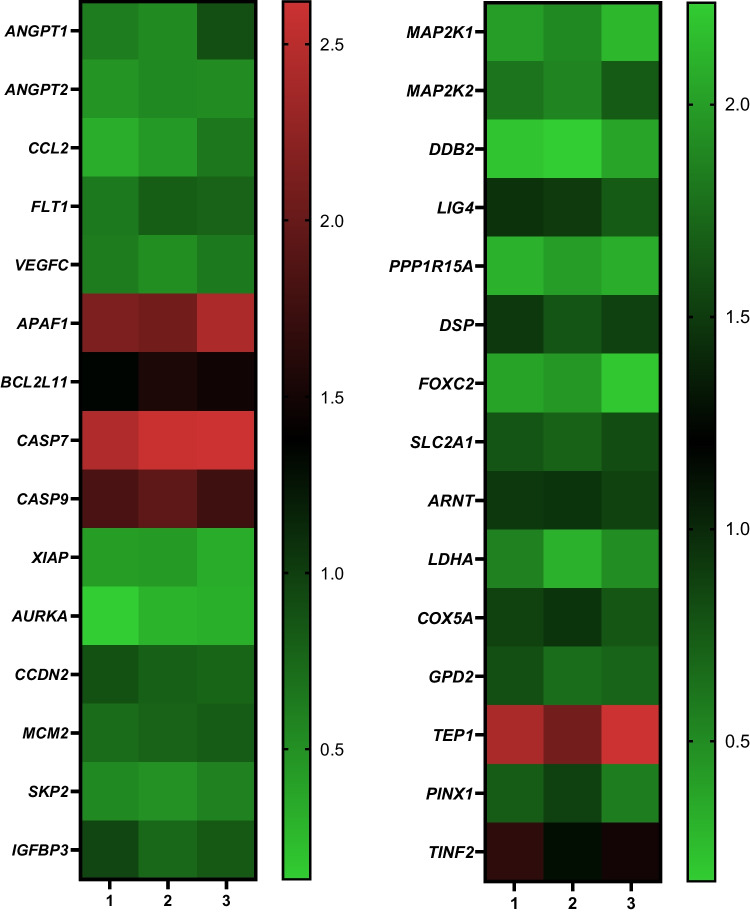
Heatmap of expression profiles of genes related with tumourigenesis in BIIB021 treated T24 cells (*n* = 3)

The expression levels of cancer-related genes were determined by IC_50_ dose of BIIB021 (16.65 nM) at the 48 h on T24 cells according to the untreated cells. The results of PCR array experiment indicated that BIIB021 in the T24 cells significantly caused the decrease/increase of the genes involved in formation and prorogation of cancer (the fold changes of genes are presented in Table 2). Furthermore, a heat map of the expression of genes in T24 cells treated with BIIB021 is presented in Fig. 4. The results demonstrated that *ANGPT1*, *ANGPT2*, *CCL2*, *FLT1*, *VEGFC*, *XIAP*, *AURKA*, *CCDN2*, *MCM2*, *SKP2*, *IGFBP3*, *MAP2K1*, *MAP2K2*, *DDB2*, *LIG4*, *PPP1R15A*, *DSP*, *FOXC2*, *SLC2A1*, *ARNT*, *LDHA*, *COX5A*, *GPD2*, and *PINX1* were downregulated, while the expressions of *APAF1*, *BCL2L11*, *CASP7*, *CASP9*, *TEP1*, and *TINF2* were increased in BIIB021 treated T24 cells.

**Table 2:** Effect of BIIB021 on expression of different genes related with tumourigenesis in T24 cells

Gene	Fold change	Function
Angiopoietin 1 (*ANGPT1*)	0.68 ± 0.19	
Angiopoietin 2 (*ANGPT2*)	0.51 ± 0.04	
Chemokine (C-C motif) ligand 2 (*CCL2*)	0.47 ± 0.17	
Fms-related tyrosine kinase 1 (vascular endothelial growth factor/vascular permeability factor receptor) (*FLT1*)	0.74 ± 0.09	Angiogenesis
Vascular endothelial growth factor C (*VEGFC*)	0.59 ± 0.07	
Apoptotic peptidase activating factor 1 (*APAF1*)	2.21 ± 0.18	
BCL2-like 11 (apoptosis facilitator) (*BCL2L11*)	1.45 ± 0.10	
Caspase 7, apoptosis-related cysteine peptidase (*CASP7*)	2.55 ± 0.09	Apoptosis
Caspase 9, apoptosis-related cysteine peptidase (*CASP9*)	1.84 ± 0.10	
X-linked inhibitor of apoptosis (*XIAP*)	0.39 ± 0.05	
Aurora kinase A (*AURKA*)	0.24 ± 0.09	
Cyclin D2 (*CCDN2*)	0.81 ± 0.06	
Minichromosome maintenance complex component 2 (*MCM2*)	0.77 ± 0.05	Cell cycle
S-phase kinase-associated protein 2 (p45) (*SKP2*)	0.54 ± 0.03	
Insulin-like growth factor binding protein 3 (*IGFBP3*)	0.84 ± 0.10	
Mitogen-activated protein kinase kinase 1 (*MAP2K1*)	0.40 ± 0.11	Cellular senescence
Mitogen-activated protein kinase kinase 2 (*MAP2K2*)	0.63 ± 0.12	
Damage-specific DNA binding protein 2 (*DDB2*)	0.25 ± 0.10	DNA damage and repair
Ligase IV, DNA, ATP-dependent (*LIG4*)	0.87 ± 0.11	
Protein phosphatase 1, regulatory (inhibitor) subunit 15A (*PPP1R15A*)	0.35 ± 0.05	
Desmoplakin (*DSP*)	0.86 ± 0.07	Epithelial-to-mesenchymal transition
Forkhead box C2 (MFH-1, mesenchyme forkhead 1) (*FOXC2*)	0.34 ± 0.12	
Solute carrier family 2 (facilitated glucose transporter), member 1 (*SLC2A1*)	0.77 ± 0.06	Hypoxia signaling
Aryl hydrocarbon receptor nuclear translocator (*ARNT*)	0.91 ± 0.04	
Lactate dehydrogenase A (*LDHA*)	0.45 ± 0.12	
Cytochrome c oxidase subunit Va (*COX5A*)	0.86 ± 0.08	Cellular metabolism
Glycerol-3-phosphate dehydrogenase 2 (mitochondrial) (*GPD2*)	0.72 ± 0.08	
Telomerase-associated protein 1 (*TEP1*)	2.03 ± 0.23	Telomeres and telomerase
PIN2/TERF1 interacting, telomerase inhibitor 1 (*PINX1*)	0.73 ± 0.16	
TERF1 (TRF1)-interacting nuclear factor 2 (*TINF2*)	1.29 ± 0.15

### Pathway analysis

**Fig. 5:**
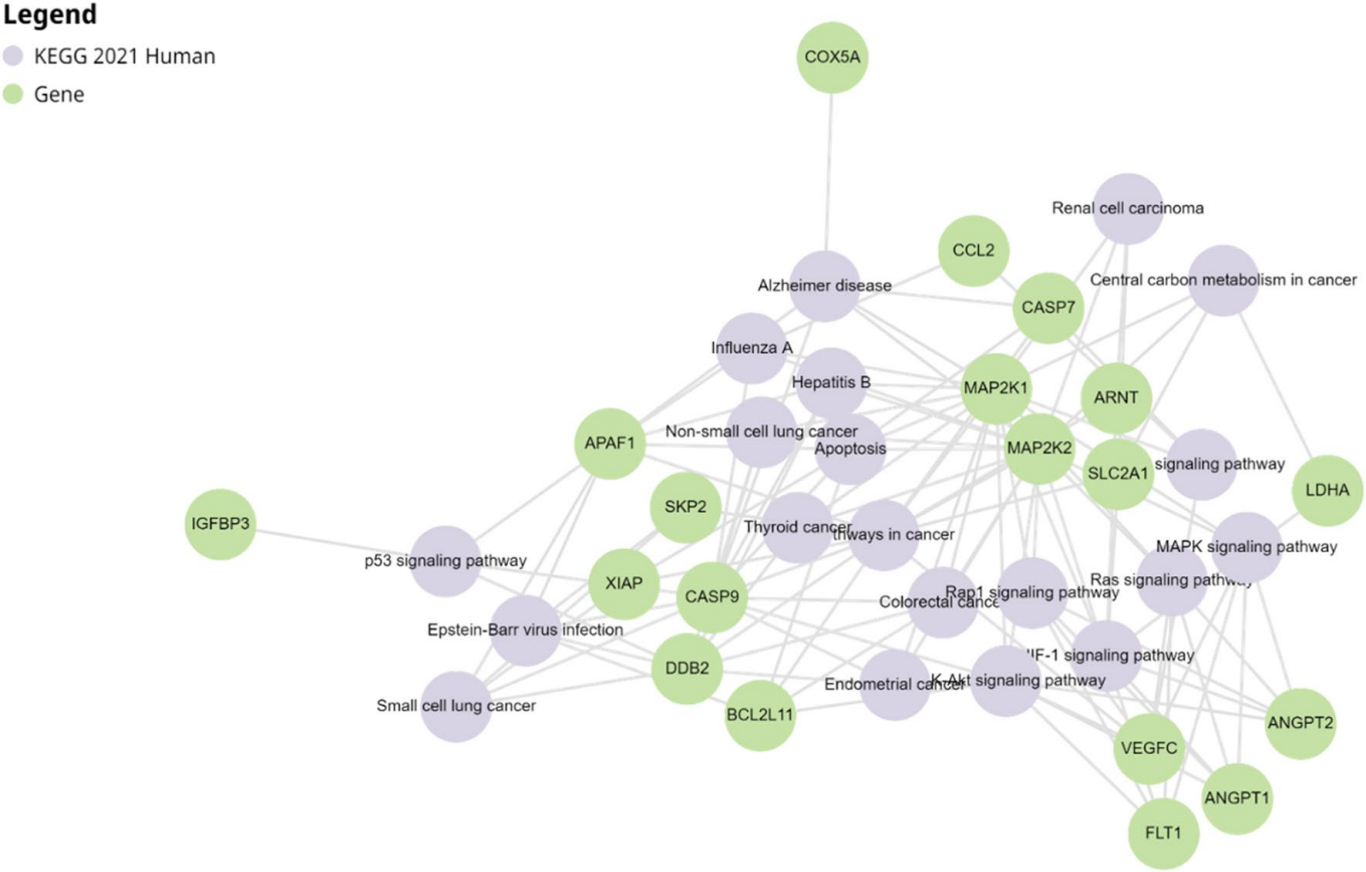
A represented network of top ten significant enriched terms in BIIB021 treated T24 cells. Pathway enrichment analysis was performed using Enrichr web tool. Thirty-one gene-set libraries were used to compute enrichment and overlap genes with pathways in KEGG database

Pathway enrichment analysis was performed with both downregulated and upregulated and genes in BIIB021 treated T24 cells using Enrichr web tool. In PCR array experiment, 31 significant down and upregulated genes were identified in effect of BIIB021 in T24 cells and the relationship of these genes with top ten important signaling pathways was determined (Table 3 and Fig. 5). The results relived that the differentially expressed genes were closely related with HIF-1 (hypoxia-inducible factor 1) signaling pathway, apoptosis, PI3K-Akt signaling pathway, colorectal cancer, small cell lung cancer, Rap1 signaling pathway, Ras signaling pathway, endometrial cancer, and renal cell carcinoma.

**Table 3:** Table of top ten significant enriched terms in BIIB021 treated T24 cells from the KEGG human gene set library. The term at the top has the most significant overlap with the input query gene set

Term	*p*-value	*q*-value	Overlap genes
HIF-1 signaling pathway	4.260695e-12	5.964973e-12	*MAP2K1, LHDA, MAP2K2, ANGPT2, FLT1, ANGPT1, SLC2A1, ARNT*
Pathways in cancer	9.666982e-12	6.766887e-12	*CASP9, MAP2K1, CASP7, MAP2K2, BCL2L11, APAF1, SLC2A1, VEGFC, ARNT, XIAP, SKP2, DDB2*
Apoptosis	1.786925e-12	8.338983e-12	*CASP9, MAP2K1, CASP7, MAP2K2, BCL2L11, APAF1, XIAP*
PI3K-Akt signaling pathway	4.922079e-12	1.722727e-12	*CASP9, MAP2K1, MAP2K2, ANGPT2, FLT1, BCL2L11, ANGPT1, VEGFC,*
Colorectal cancer	2.033154e-12	5.692831e-12	*CASP9, MAP2K1, MAP2K2, BCL2L11, DDB2*
Small cell lung cancer	2.852333e-12	6.655445e-12	*CASP9, APAF1, XIAP, SKP2, DDB2*
Rap1 signaling pathway	7.378379e-12	1.475676e-12	*MAP2K1, MAP2K2, ANGPT2, FLT1, ANGPT1, VEGFC*
Ras signaling pathway	1.319096e-12	2.308420e-12	*MAP2K1, MAP2K2, ANGPT2, FLT1, ANGPT1, VEGFC*
Endometrial cancer	1.889343e-12	2.938978e-12	*CASP9, MAP2K1, MAP2K2, DDB2*
Renal cell carcinoma	3.804308e-12	4.769081e-12	*MAP2K1, MAP2K2, SLC2A1, ARNT*

## Discussion

In this study, the molecular mechanisms of anticancer effect of BIIB021 were investigated in human bladder cancer cell line. BIIB021 exhibited inhibitory effect on T24 cell viability in a dose- and time-dependent manner, and the IC_50_ value of BIIB021 was calculated as 16.65 nM at 48 h. In the literature, anticancer activities of different HSP90 inhibitors were examined in bladder cancer cell lines. 17-DMAG (17-Dimethylaminoethylamino-17-demethoxygeldanamycin) is a semi-synthetic and water soluble HSP90 inhibitor that decreased the viability of T24, UM-UC-3, and 5637 cells at nM concentrations (IC_50_ values for T24, UM-UC-3 and 5637 cells were calculated as 316 nM, 471 nM and 32 nM at 48 h, respectively) (Yoshida et al. 2011). In another experimental study, the antiproliferative potentials of second generation HSP90 inhibitors (AUY922 (luminespib), STA-9090 (ganetespib), SNX2112, AT13387 (onalespib), and CUDC305) were evaluated against in bladder carcinoma 5637 cells. These inhibitors inhibit ATPase function of HSP90, and block cancer-related signaling pathways via degradation of oncogenic client proteins. AUY922, STA-9090, SNX2112, AT13387, and CUDC305 exhibited inhibitor effect on the cell proliferation of 5637 cells at 48 h with the IC_50_ values of 2.21 nM, 18.50 nM, 26.30 nM, 9.56 nM, and 344 nM, respectively (Li et al. 2017). Compared to these results, BIIB021 showed similar anticancer effect in T24 cells at lower concentrations. In previous clinical study, anticancer activity of BIIB021 was evaluated in patients with gastrointestinal stromal tumor. The mean C_max_ value of BIIB021 was determined as about 1.5 µM after oral administration. C_max_ is defined as the maximum plasma concentration value of drug that provides information about how the body will react to the drug (Dickson et al. 2013). Calculated C_max_ value exceeds the IC_50_ concentrations of BIIB021 in T24 cells. In clinical applications, BIIB021 can lead to different metabolic responses in patients and the dose of the drug may be administered at a level higher than the IC_50_ value (Dickson et al. 2013). IC_50_ values of BIIB021 at nM level in T24 cells appear to be a promising result for clinical studies.

Our results indicated that BIIB021 decreased protein expression of HSP90 and the refolding efficiency of denatured luciferase in T24 cells. Aberrant expression of HSP90 activates stabilization and proper folding of client proteins, resulting in stimulation of the progression of cancer cells. Therefore, cancer cells are sensitive to decrease in chaperone activity of HSP90 due to destabilization and disruption of correct folding of client proteins (Özgür et al. 2021; Karagöz and Rüdiger 2015). In this study, luciferase was used as a model substrate for investigation of inhibitory effect of BIIB021 on HSP90 chaperone activity. The coordination of HSP90 and other chaperone proteins results in the refolding of denatured luciferase in vitro conditions. BIIB021 inhibits the refolding activity of denatured luciferase in the presence of T24 bladder cancer cell lysates and recombinant HSP90. Several studies reported that BIIB021 and some HSP90 inhibitors decreased the refolding activity of denatured luciferase in several cancer cell lines. Güven et al. indicated that the addition of BIIB021 (14.79 nM) in reaction mixture significantly decreased the refolding ratio of luciferase in human cervical adenocarcinoma cell line (HeLa) (Güven and özgür 2023). In another study, 15.17 nM of STA-9090 decreased HSP90 expression level (1.71-fold) and luciferase refolding activity by 2.04-fold in HeLa cells (Karademir and Özgür 2023).

To understand the molecular mechanism of the anticancer activity of BIIB021 in bladder cancer cells, the mRNA expressions of the cancer-related genes were investigated by PCR array experiments. *ANGPT1*, *ANGPT2*, *CCL2*, *FLT1*, and *VEGFC* are five important angiogenic genes that play significant roles in blood vessel stabilization and metastases of cancer cells (Kiss and Saharinen 2019; Elayat et al. 2023). *ANGPT1* and *ANGPT2* are overexpressed in cancer cells, and they contribute to development and progression of cancer. *ANGPT1* is crucial for newly formed blood vessels whereas *ANGPT2* destabilizes existing blood vessels for the generation of new blood vessels in collaboration with *VEGF* (Huang et al. 2010). In cancer cells, aberrant expression of *CCL2* promotes angiogenesis with activation of proliferation, migration, and tube formation in human umbilical vein endothelial cells (Peng et al. 2022). *FLT1* stimulates tumor growth and metastasis, and it is an important biological target for antiangiogenic drug discovery (Shibuya 2011). *VEGFC* is a key regulator of angiogenesis, and its overexpression promotes tumor invasion and metastasis (Su et al. 2007). Therefore, the decrease in the expression of *ANGPT1*, *ANGPT2*, *CCL2*, *FLT1*, and *VEGFC* genes plays an important role in preventing metastasis of cancer cells (Cardones and Banez 2006; Jayson et al. 2016). In this study, BIIB021 reduced the expressions of *ANGPT1*, *ANGPT2*, *CCL2*, *FLT1*, and *VEGFC* genes, and it has a big potential as an antiangiogenic agent in T24 bladder cancer cells.

Our results demonstrated that the expressions of *BCL2L11, APAF1*, *CASP7*, and *CASP9* were upregulated, whereas the expressions of *XIAP* were downregulated after the treatment of T24 cells with BIIB021. The formation and progression of cancer are characterized by the suppression of apoptotic pathways. *APAF1*, *CASP7*, *CASP9*, *BCL2L11,* and *XIAP* are important members of the intrinsic apoptotic pathway (mitochondrial pathway of apoptosis). BCL2L11 activates the releasing of cytochrome-c (CYT-c) from mitochondria. In the cytosol, CYT-c interacts with APAF1, resulting in the formation of the apoptosome complex. Then, CASP9 binds to the apoptosome complex, and apoptosis is stimulated with activation of CASP3, CASP6, and CASP7. On the other hand, XIAP inhibits caspase activation and mitochondrial CYT-c release (Jendrossek 2012; Elmore 2007; Abbas and Larisch 2020). It is well-known that the overexpression of HSP90 inhibits apoptotic pathways to stimulate cancer cell proliferation. Numerous studies have indicated HSP90 inhibitors suppress cell growth by inducing apoptosis in cancer cells (Wang et al. 2014a, b; Jego et al. 2013). Güven et al. proved that BIIB021 (14.79 nM) activated intrinsic apoptotic pathways by the upregulation of pro-apoptotic markers (BAX, CASP3, CASP9, and CYT-c) in the treated HeLa cervical cancer cells (Güven and Özgür 2023). Georgakis et al. indicated that the low dose of 17-AAG (first-generation HSP90 inhibitor) activates CASP3, CASP9, and important pro-apoptotic markers (BAX and BID) in mantle cell lymphoma cell lines, resulting in stimulation of intrinsic apoptotic pathway (Georgakis et al. 2006). In another experimental study, Debio‑0932 (oral HSP90 inhibitor) increased the expression of BAX and CASP9, and whereas decreased BCL-2 expression in human breast cancer cell line, MCF-7 and MDA-MB-231 (Özgür et al. 2021). Our findings indicated that the cytotoxicity effect of BIIB021 against T24 cells may explain with activation of intrinsic apoptotic pathway.

In normal cells, *AURKA*, *CCDN2*, *MCM2*, and *SKP2* genes have vital roles in the regulation of the mitosis, cell cycle, and histone modification. These genes are overexpressed in different cancer types and related to malignant progression and resistance to chemotherapy (Du et al. 2021; Knudsen et al. 2022). Che et al. demonstrated that HSP90 inhibitor SNX-7081 inhibited DNA replication and repair mechanisms by decreasing *CCDN2* and *MCM2* expressions in human chronic lymphocytic leukemia cells (Che et al. 2013). Wang et al. reported that 17-AAG and 17-DMAG stimulated cell differentiation by degradation of AURKA in a MPLW515L mouse model of primary myelofibrosis (Wang et al. 2023). We showed that BIIB021 was reduced the gene expressions of *AURKA*, *CCDN2*, *MCM2*, and *SKP2* in T24 cells. BIIB021 may act as a mitosis and cell cycle inhibitor in bladder cancer cells, resulting in prevention of uncontrolled cell division.

The expression of *IGFBP3*, *MAP2K1*, and *MAP2K2* has been closely linked to the pathogenesis of cancers. Abnormal expression of *IGFBP3* is also involved in tumor progression and metastases in a wide variety of cancer cells (Mizuno et al. 2023; Cai et al. 2020). On the other hand, IGFBP3 has an antioxidative activity and stimulates intrinsic apoptotic pathways with the reducing reactive oxygen species (ROS) levels in certain cancer types (Wang et al. 2021). In the literature, the biological roles of *IGFBP3* in bladder cancer cells have not been understood yet. In current study, BIIB021 reduced the gene expression level of T24 cells and the low *IGFBP3* level may prevent proliferation of bladder cancer cells. *MAP2K1* and *MAP2K2* are specific kinases that activate the mitogen-activated protein kinase (MAPK) pathway. Abnormal activation of MAPK signaling pathway may lead to increased cell proliferation and resistance to apoptosis in cancer cells. Therefore, the downregulation of *MAP2K1* and *MAP2K2* expression prevents cancer cell survival (Burotto et al. 2014). Our results demonstrated that the gene expression of *MAP2K1* and *MAP2K2* was decreased in BIIB021 treated T24 bladder cancer cells.

Several experimental studies reported that *DDB2*, *LIG4*, and *PPP1R15A* are involved in DNA damage and repair mechanisms in cells (Majercikova et al. 2022; Tomkinson et al. 2020). *DDB2* acts either as an oncogene or a tumor suppressor gene. *DDB2* indirectly facilitates DNA replication and G1/S transition. In many cancer types, the activation of *DDB2* attenuates the detoxification of ROS that stimulates several signaling pathways involved in tumor formation. Furthermore, *DDB2* facilitates the repair mechanisms of DNA double-strand breaks after radiation therapy and inhibits apoptotic pathways in cancer cells (Gilson et al. 2019). The association between cancer formation and the expressions of *LIG4* and *PPP1R15A* was identified in several in vitro studies. The abnormal expressions of *LIG4* and *PPP1R15A* trigger cancer cell proliferation via activation of DNA repair pathways (Majercikova et al. 2022). Here, the gene expressions of *DDB2*, *LIG4*, and *PPP1R15A* were decreased in BIIB021 treated bladder cancer cells. We demonstrated that BIIB021 was downregulated DNA repair related genes to prevent bladder cancer cell proliferation.

The epithelial-to-mesenchymal transition (EMT) is a vital biological process for embryonic development, tissue regeneration, organ fibrosis, and wound healing in normal physiological conditions. In cancer cells, EMT is involved in tumorigenesis, metastases, the formation of tumor cells with stem cells, and resistance to chemotherapy (Francou and Anderson 2020; Dudas et al. 2020). *DSP* and *FOXC2* genes are significant actors in EMT, and their upregulation promotes tumorigenesis. Therefore, the inactivation of *DSP* and *FOXC2* is an important therapeutic target for cancer treatment (Hader et al. 2010; Zhou et al. 2017). Our findings indicated that BIIB021 decreased *DSP* and *FOXC2* expressions in T24 cells and blocks EMT process.

Hypoxia changes cancer cell metabolism and stimulates the production of ROS, resulting in tumor cell survival and progression (Sebestyén et al. 2021). *SLC2A1*, *ARNT*, and *LDHA* are important members of the hypoxia signaling pathways and the alteration of their expressions regulates tumorigenesis. Previous studies manifested that the level of *SLC2A1*, *ARNT*, and *LDHA* is upregulated in many human cancer types (Ooi and Gomperts 2015; Huang et al. 2015). In the present investigation, *SLC2A1*, *ARNT*, and *LDHA* were downregulated in BIIB021 treated T24 cells for inhibition of hypoxia signaling pathways.

In this report, important metabolism genes, *COX5A* and *GPD2*, were investigated in BIIB021 treated bladder cancer cells and were found to be statistically downregulated. *COX5A* and *GPD2* expressions are significantly increased in cancer cells and may inhibit the apoptotic signaling pathways and protected cancer cells against chemotherapy-induced oxidative stress (Majercikova et al. 2022; Tran et al. 2022).

Human telomeres become shorter in cell division process, resulting in stimulation of apoptosis and cellular senescence (Artandi and DePinho 2010). *PINX1* interacts with catalytic activity of telomerase and inhibits telomerase activation. It is worthwhile to note that the abnormal expression of *PINX1* is associated with poor prognosis and promotes cancer cell proliferation (Li et al. 2016). *TEP1* and *TINF2* play important roles in telomere maintenance processes and act as a tumor suppressor in cancer cells (Yin et al. 2020). Our PCR array results indicated that BIIB021 was downregulated *PINX1* expression, whereas upregulated *TEP1* and *TINF2* expressions in bladder cancer cells.

Pathway enrichment analysis was performed for identifying disease-risk pathways according to down and up-regulated genes in BIIB021 treated T24 cells. The regulation of this gene set in effect of BIIB021 is related with HIF-1 signaling pathway, apoptosis, PI3K-Akt signaling pathway, colorectal cancer, small cell lung cancer, Rap1 signaling pathway, Ras signaling pathway, endometrial cancer, and renal cell carcinoma. Especially, HIF-1 signaling pathway is closely associated with the regulation of gene set in BIIB021 treated T24 cells. HIF-1 signaling pathway plays crucial roles in tumorigenesis, and regulates metastasis, angiogenesis, cancer cell proliferation, and drug resistance in cancer cells (Masoud and Li 2015; Infantino et al. 2021). The regulation HIF-1 signaling pathway with BIIB021 may prevent tumorigenesis in bladder cancer cells.

## Conclusions

In this study, BIIB021 exhibited cytotoxic effect on T24 human bladder cancer at nanomolar concentrations. The protein expression of HSP90 in T24 cells and the refolding activity of denatured luciferase were reduced in effect of BIIB021. Finally, BIIB021 was regulated the expression of metastases, apoptosis, cell cycle, cellular senescence, DNA damage and repair, EMT, hypoxia, cellular metabolism, and telomerase-related genes in bladder cancer cells. Particularly, BIIB021 inhibited cell viability of bladder cancer cells via stimulation of apoptosis, and its antiangiogenic potential in T24 cells was determined via gene expression study. Our findings indicated that BIIB021 is a potent HSP90 inhibitor to prevent bladder cancer formation and progression.

## Data Availability

No datasets were generated or analysed during the current study.
